# PARAGEN 1.0: A Standardized Synthetic Gene Library for Fast Cell-Free Bacteriocin Synthesis

**DOI:** 10.3389/fbioe.2019.00213

**Published:** 2019-09-06

**Authors:** Philippe Gabant, Juan Borrero

**Affiliations:** Syngulon, Seraing, Belgium

**Keywords:** antimicrobial peptides, bacteriocins, cell-free extracts, *in vitro* synthesis, synthetic biology

## Abstract

The continuous emergence of microbial resistance to our antibiotic arsenal is widely becoming recognized as an imminent threat to global human health. Bacteriocins are antimicrobial peptides currently under consideration as real alternatives or complements to common antibiotics. These peptides have been much studied, novel bacteriocins are regularly reported and several genomic databases on these peptides are currently updated. Despite this, to our knowledge, a physical collection of bacteriocins that would allow testing and comparing them for different applications does not exist. Rapid advances in synthetic biology in combination with cell-free protein synthesis technologies offer great potential for fast protein production. Based on the amino acid sequences of the mature peptide available in different databases, we have built a bacteriocin gene library, called PARAGEN 1.0, containing all the genetic elements required for *in vitro* cell-free peptide synthesis. Using PARAGEN 1.0 and a commercial kit for cell-free protein synthesis we have produced 164 different bacteriocins. Of the bacteriocins synthesized, 54% have shown antimicrobial activity against at least one of the indicator strains tested, including Gram-positive and Gram-negative bacteria representing commonly used lab strains, industrially relevant microorganisms, and known pathogens. This bacteriocin collection represents a streamlined pipeline for selection, production, and screening of bacteriocins as well as a reservoir of ready-to-use antimicrobials against virtually any class of relevant bacteria.

The remarkable health benefits that have been achieved with antibiotics are being questioned by the rapid increase of resistant bacteria. World Health Organization's new Global Antimicrobial Surveillance System (GLASS) study shows an antibiotic resistance occurrence among 500,000 people with suspected bacterial infections throughout 22 countries (WHO, 2015). Hence a growing list of infections like pneumonia, tuberculosis and foodborne diseases are becoming sometimes impossible to treat as antibiotics are not so effective (WHO, 2015). The antibiotic resistance crisis has been attributed to an excessive as well as abusive use of antibiotics, and also as the consequence in the decrease of new drug development by the pharmaceutical industry due to reduced economic incentives and demanding regulatory requirements (Cooper and Shlaes, 2011; White, 2011). Moreover, it has been shown that the consumption of broad-spectrum antibiotics may damage the human commensal microbiota which has major roles in the host's health (Blaser, 2011; Willing et al., 2011; Lange et al., 2016). Consequently, the development of new antimicrobial agents that may be used in different settings (clinic, food industry, animal feed, etc.) has become crucial.

Antimicrobial peptides (AMPs) have been described as “evolutionarily ancient weapons against microbial infections” (Zasloff, 2002). AMPs are produced by all organisms, including human beings, and play an essential role in innate immunity. They are key elements of the innate immune system which deliver immediately effective and non-specific defenses against infections. Among the large variety of AMPs, there is a subgroup known as bacteriocins which are small peptides, produced by bacteria and ribosomally synthesized. Bacteriocins, which were discovered in 1925 (Gratia and Fredericq, 1946), exhibit strong activity against their target strains. This activity has often been found in the nanomolar range, making them in some cases more potent than their antibiotic analogs (Mathur et al., 2013; Ming et al., 2015). Another interesting feature is that, compared to antibiotics, most bacteriocins are only active against a narrow spectrum of bacteria close related to the producer. A few years ago, this narrow inhibition spectrum was considered as a major bottleneck, however today we know that big disruptions in the gut microbiota caused by broad spectrum antibiotics are coupled to immunological, metabolic, and neurological disorders in the host. Therefore, it is believed that targeting specific bacteria instead of the whole microbiome is more efficient and less intrusive (Mills et al., 2017).

Bacteriocins are an heterogenous group with a wide range of sizes, structures, modes of action, activity spectra, and target cell receptors. Their classification undergoes continuous modifications because of new developments regarding their structures and modes of action but they may be broadly divided into class I (post-translationally modified) class II (heat-stable, <10 kDa, unmodified) and class III (thermo-labile, >10 kDa) groups (Álvarez-Sieiro et al., 2016). Along with categorizing bacteriocins based on their modifications, they may be subdivided. The unmodified (class II) bacteriocins can be divided into four groups according to the classification by Álvarez-Sieiro et al. (2016). These subclasses are peptides that contain a YGNGV motif (in which N represents any amino acid; the class IIa peptides); two-peptide bacteriocins (class IIb peptides); leaderless bacteriocins (class IIc peptides); and unmodified, linear, non-pediocin-like, single-peptide bacteriocins that do not belong to other subclasses (class IId peptides).

Since the early 2000s, the successfulness of bacteriocins against unwelcome microorganisms has opened unumbered opportunities for innovative research, and currently, research on these AMPs is rapidly gaining importance. Bacteriocins on their own have shown a great potential mainly in food preservation and biomedical applications, and as a matter of fact, over 60% of the bacteriocin-related patents granted are related to these two areas. However, the use of bacteriocins in other alternative fields is growing, and there are many examples of bacteriocins for veterinary use, cosmetic use and bacteriocins applied on production–purification systems along with recombinant proteins or molecular modifications in the producer strains (López-Cuellar et al., 2016).

Since 2008, pharmaceutical companies have only introduced four new antibiotics, compared to the 16 approved from 1983 to 1987 and, for more than 40 years there have been no releases of new classes of antibiotics to treat Gram-negative bacilli. Meanwhile, the number of new bacteriocins is rapidly increasing and it is believed that more than 99% of bacteria can produce at least one bacteriocin, although most of which are still not identified (Riley and Wertz, 2002). Only from year 2004 to 2015, there have been published 429 scientific articles and 245 granted patents related to bacteriocins and these numbers are increasing to almost one bacteriocin related publication per day (López-Cuellar et al., 2016). In an effort to centralize all this information, there are several bacteriocin sequence data repository websites (http://bactibase.hammamilab.org/main.php, http://bagel4.molgenrug.nl/) regularly updated with the most relevant features of the new bacteriocins described. Moreover, in the last years there has been an increasing number of web servers hosting bioinformatic software acting as bacteriocin genome mining tools allowing the research community to identify bacteriocins and other antimicrobial peptides in genome/metagenome sequences (http://bactibase.hammamilab.org/main.php, http://bagel4.molgenrug.nl/, https://antismash.secondarymetabolites.org/#!/start). Despite a clear interest in exploring the full potential of these peptides and the enormous amount of information on bacteriocins provided by researcher groups from all over the world, application of their potential remains to be explored. As with other molecules, the more information there is, the more difficult it is to choose the right candidate for a specific application.

We have identified that the availability of a physical collection of bacteriocin genes designed in a standardized format allowing a rapid production of peptides would boost innovation in the AMPs research community. This new resource should also allow for the first time to test and compare different bacteriocins, make combinations to analyse and characterize potential synergistic effects between unrelated bacteriocins as well as compare biochemical properties. Academic and industrial researchers would be able then to choose and select the right bacteriocin from this collection, tailored for each specific application.

Bacteriocin production and purification is not an easy task and is considered as one of the main bottlenecks for their use at industrial scale (Deraz et al., 2007; Jozala et al., 2008, 2015; Bali et al., 2014). Bacteriocins traditionally have been purified from their native producing strains, however this consists in a time-consuming and laborious procedure which often results in low yields of bacteriocin (Rodríguez et al., 2003). On top of that, collecting all bacteriocin producing strains described and purifying their active peptides is an almost impossible task. Other methods such as the heterologous production of bacteriocins in other microorganisms (bacteria or yeast) and in plants has been shown as a useful technology for producing higher concentrations of these peptides (Sánchez et al., 2008; Borrero et al., 2011, 2018). Nevertheless, only a few bacteriocins have been successfully produced by heterologous hosts since this approach is laborious and time consuming. Moreover, the risk that the bacteriocin will be produced at a yield not reaching the standard request of industrial processes is still quite high. There are alternatives to the use of microorganisms as peptide producers and with the recent advances in the field of Synthetic Biology, it is now possible to synthesize peptides both by chemical synthesis and by using cell-free extracts for *ex-vivo* protein production (Hols et al., 2019). Since its first application in deciphering genetic codes (Nirenberg and Matthaei, 1961) cell-free protein synthesis (CFPS) has become a key method for the obtention of recombinant proteins in order to match the rising demands for simple, inexpensive, and efficient protein production (Carlson et al., 2012; Hodgman and Jewett, 2012). The recent growing attention to crude extract CFPS is motivated mainly, but not only, by the unique benefit that barrier-free cell-free systems enable direct and fast access to enzymes and reaction conditions.

The peptidic nature of bacteriocins and the fact that they are ribosomally synthesized (Field et al., 2012) make them ideal candidates for production by CFPS. With the cell-free system there is no need of signal peptides or leader sequences attached to the bacteriocins and of dedicated transporter systems to secrete the mature and active peptide, as the bacteriocin is synthesized outside the cell. Moreover, with this technology the potential toxicity to the host is also excluded and there is no need to coproduce the immunity protein. This major technological locket could be overcome in most cases by working with cell-free extracts which is by design an abiotic technology. With these advantages in mind we have constructed a physical collection of bacteriocins: PARAGEN 1.0: a standardized synthetic gene library allowing to produce a broad set of bacteriocins in parallel.

A schematic description of the methodology used is shown in Figure 1. A list of class II bacteriocins was obtained from the two major genomic bacteriocin databases: BAGEL4 (van Heel et al., 2018) and BACTIBASE (Hammami et al., 2010). The aminoacidic sequence of the mature/active peptide of each bacteriocin was determined based on the information provided by these databases and, in some cases, going to the original publications and/or sequences found at the NCBI database (Database resources of the National Center for Biotechnology Information, 2015). All aminoacidic sequences were reverse-translated and codon optimized for *Escherichia coli* (www.bioinformatics.org/sms2/rev_trans.html). The nucleotide sequences were included in a vector backbone containing the T7 promoter region, a start codon (ATG) a stop codon (TAA) and a T7 terminator region. Recombinant vectors were constructed and amplified in *E. coli* DH10B standard strain and used as templates for cell-free protein synthesis using PURExpress® *in vitro* Protein Synthesis Kit (New England Biolabs). All bacteriocins synthesized were tested on petri dishes for antimicrobial activity against a set of 12 different indicators including Gram-positive and Gram-negative bacteria (Table 1). Bacteriocins showing a halo of inhibition against at least one of the indicators were considered as positive (Supplementary Material). A total of 128 bacteriocins were synthesized, 23 belonging to the class IIa (18%), 15 belonging to the class IIb (12%), 15 belonging to the class IIc (12%), 50 belonging to the class IId (39%), and 25 belonging to the class III (20%) (Figure 2). Regarding their antimicrobial activity, 74% of the class IIa, 67% of the class IIb, 100% of the class IIc, 38% of the class IId and 32% of the class III bacteriocins showed activity against at least one of the indicators tested. The remaining bacteriocins (59 out of 128) did not show activity against any of the indicators tested (Supplementary Material). However, we cannot conclude that these 59 bacteriocins are inactive as, most likely, they are just not active against the 12 indicators used in this work. Hence, a further analysis with a larger number of targets should be done. Other tests such as protease and pH stability of these peptides are currently being done in the lab.

**Figure 1:**
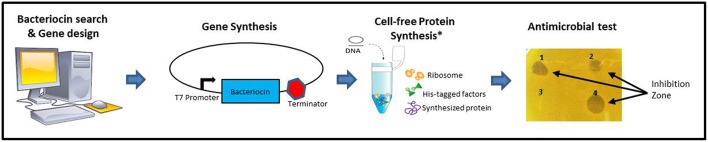
Schematic description of the methodology used in the construction of PARAGEN 1.0. *Image from PURExpress® *in vitro* Protein Synthesis Kit (New England Biolabs).

**Table 1:** Strains used as indicators to test the antimicrobial activity of the bacteriocins from PARAGEN 1.0.

**Strain**	**Gram**
*Escherichia coli* DH10B	–
*Bacillus cereus* ATCC 14579	+
*Bacillus subtilis* 168	+
*Enterococcus faecalis* Si0159	+
*Enterococcus faecium* ATCC 19434	+
*Lactococcus lactis* IL1403	+
*Listeria monocytogenes* ATCC 19115	+
*Pediococcus pentosaceus* HELA	+
*Staphylococcus aureus aureus* ATCC 6538	+
*Staphylococcus epirdermidis* ATCC 12228	+
*Streptococcus mutans* UA159	+
*Streptococcus pyogenes* ATCC 12344	+

**Figure 2:**
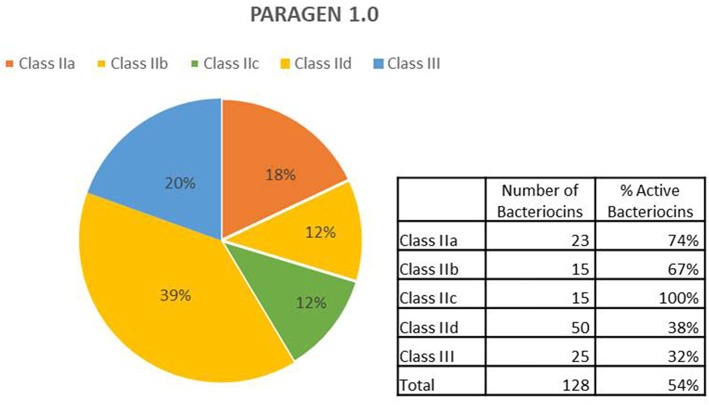
Description of bacteriocins in PARAGEN 1.0. From the total, 18% are class IIa peptides; 12% are considered class IIb, 12% belong to class IIc, 39% are class IId and 20% are class III bacteriocins. Among bacteriocins from class IIa, 74% were active; 67% from class IIb were active, all bacteriocins from class IIc were active (100%), 38% and 32% of bacteriocins from class IId and class III, respectively, showed antimicrobial activity against one or more indicators.

As shown in this work, synthetic production of bacteriocins with cell-free systems is a viable alternative to bacterial production as it allows very rapidly the synthesis and screening of a large numbers of bacteriocins in the same experiment. Further efforts need to be done in order to reduce the cost of this system, optimize and increase peptide production. There is also a need on finding a method to quantify the production of active bacteriocin in order to fully compare antimicrobial activities. Several authors have already used cell-free as platform for the production for both single proteins but also for proteins produced through complex metabolic pathways (Koch et al., 2018). Some class I bacteriocins such as microcin J25 have been modified and produced in their active conformation *ex-vivo* (Yan et al., 2012), therefore we strongly believe that the production of class I bacteriocins should be possible soon with this technology.

To our knowledge, PARAGEN 1.0 is the largest collection of active native bacteriocins ready to be tested and it keeps growing since we are constantly adding new peptides to it. We are already working in a second library of non-natural bacteriocins (PARAGEN 2.0) consisting on chimeras of different bacteriocins or variants with single or multiple amino acid modifications. The possibilities that cell-free systems offer for bacteriocin production are almost unlimited and we believe that PARAGEN might be a useful tool for gaining knowledge of the precise modes of action of bacteriocins and more generally antimicrobial peptides, allowing the production of more active and more stable variants. This would contribute to speed up the screening for natural antimicrobial activities and the design of tailor-made bacteriocins for their applications in food industries and in medicine.

## Data Availability

All datasets generated for this study are included in the manuscript/Supplementary Files.

## Author Contributions

PG and JB have contributed equally to the design and implementation of the research, to the analysis of the results and to the writing of the manuscript.

### Conflict of Interest Statement

PG and JB were employed by company Syngulon S.A.
