# Supplementary material for: PARAGEN 1.0: A Standardized Synthetic Gene Library for Fast Cell-Free Bacteriocin Synthesis
Source: Front Bioeng Biotechnol. 2019 Sep 6;7:213. doi: 10.3389/fbioe.2019.00213 (PMC6743375; doi:10.3389/fbioe.2019.00213)
Supplement: Supplementary file 1 [file Table_1.DOCX]

**SUPPLEMENTARY MATERIAL**

Bacteriocins synthesized *in vitro* for PARAGEN 1.0

| **Bacteriocin** | **Class** | **Size in AA** | **Spectrum of Activity^a,b^** | **Reference^c^** |
| --- | --- | --- | --- | --- |
| Acidocin A | IIa | 58 |  | [1] |
| Avicin A |  | 43 | LIS, EF, EFL | [2] |
| Bacteriocin 43 |  | 44 | LIS, EF, PP | [3] |
| Bacteriocin J46 |  | 27 |  | [4] |
| Carnobacteriocin BM1 |  | 44 | LIS, EF, EFL | [5] |
| Enterocin 35 |  | 44 | PP | [6] |
| Enterocin A |  | 47 | LIS, EF | [7] |
| Enterocin E50-52 |  | 39 | LAC | [8] |
| Enterocin E760 |  | 51 |  | [9] |
| Leucocin A |  | 37 | LIS, PP | [10] |
| Leucocin C |  | 43 | EF, EFL | [11] |
| Mundticin L |  | 43 | EF, EFL | [12] |
| Pediocin PA-1 |  | 44 | LIS, EFL, PP | [13] |
| Pediocin PA1 (1-7) / Enterocin E50-52 (10-39) |  | 37 |  | [14] |
| Piscicolin 126 |  | 44 | EF, EFL | [15] |
| Plantaricin 423 |  | 37 | LIS | [16] |
| Sakacin A |  | 41 | EF, EFL | [17] |
| Sakacin G |  | 38 |  | [18] |
| Sakacin P |  | 43 |  | [19] |
| Sakacin P (1-17) / Enterocin A (23-47) |  | 42 | PP | [20] |
| Sakacin P (1-17) / Pediocin PA1 (18-45) |  | 44 | PP | [20] |
| Sakacin X |  | 43 | EF, EFL | [21] |
| Ubericin A |  | 49 | LAC, LIS, EF, SP, PP | [22] |
| Abp118 (α+ß) | IIb | 45+46 | LAC, LIS, EF, SP, PP | [23] |
| Amylovorin L (α+ß) |  | 62+50 |  | [24] |
| Brochocin C (α+ß) |  | 59+43 | LIS, EF, SP, PP | [25] |
| Lactacin F (A+X) |  | 57+48 | LAC, SA, EF, EFL | [26] |
| Lactocin 705 (α+ß) |  | 33+33 |  | [27] |
| Lactococcin G (α+ß) |  | 39+35 | LAC, LIS, EF | [28] |
| Lactococcin Q (α+ß) |  | 39+35 | LAC, EF | [29] |
| Plantaricin EF (E+F) |  | 33+34 | LAC, SP | [30] |
| Plantaricin JK (J+K) |  | 25+32 |  | [30] |
| Plantaricin NC8 (α+ß) |  | 29+34 | EFL | [31] |
| Plantaricin S (α+ß) |  | 27+27 | LAC | [32] |
| Plantaricin W (α+ß) |  | 29+33 |  | [33] |
| Sakacin T (α+ß) |  | 51+43 | EF, EFL | [21] |
| **Bacteriocin** | **Class** | **Size in AA** | **Spectrum of Activity^a,b^** | **Reference^c^** |
| Thermophilin 13 (α+ß) | IIb | 62+43 | LAC, EF, SP | [34] |
| Thuricin CD (α+ß) |  | 30+30 |  | [35] |
| Aureocin A53 | IIc | 51 | LAC, SA, EF, EFL, PP | [36] |
| Aureocin A70 (A+B+C+D) |  | 31+30+31+31 | LAC, SA, LIS, SE, EFL | [37] |
| Cereucin H (A+B+C) |  | 26+30+30 | LAC | [38] |
| Cereucin V (A+B+C) |  | 30+30+31 | LAC, LIS | [38] |
| Cereucin X (A+B+C) |  | 27+29+30 | LAC, SA, EF, EFL | [38] |
| Enterocin 7 (A+B) |  | 44+43 | LAC, SA, LIS, EF, EFL | [39] |
| Enterocin L50 (A+B) |  | 44+43 | EC, LAC, SA, LIS, EF, SP, EFL, PP | [40] |
| Garvicin KS - (A+B+C) |  | 34+34+32 | LAC, SA, LIS, EF, SE, EFL | [38] |
| Lacticin Q |  | 53 | LAC, SA, LIS, EF, EFL | [41] |
| Lacticin Z |  | 53 | LAC, SA, LIS, EF, EFL | [42] |
| Lacticin Z - Variant 1 |  | 48 | LAC, LIS | WP_046945355.1 |
| Lacticin Z- Variant 2 |  | 48 | LAC, SA, EF, EFL | WP_078984773.1 |
| Mutacin BHT-B |  | 44 | LAC, EF, EFL | [43] |
| Weissellicin M |  | 43 | LAC, EF | [44] |
| Weissellicin Y |  | 43 | LAC | [44] |
| Acidocin 8912 | IId | 26 |  | [45] |
| Bacteriocin 32 |  | 70 | EF | [46] |
| Bacteriocin BlpK_ss |  | 52 | LAC, EF, EFL, PP | WP_013991125.1 |
| Bacteriocin UviB |  | 76 |  | [47] |
| Bactofencin A |  | 22 | SE | [48] |
| Cerein 7B |  | 56 | LAC | [49] |
| Colicin V (Microcin C) |  | 88 | EC | [50] |
| Enterocin EJ97 |  | 44 | SA, EF, EFL | [51] |
| Enterocin K1 |  | 38 | EF, EFL | [52] |
| Epidermicin Ni01 |  | 51 | LAC, EF, EFL | [53] |
| Garvieacin Q |  | 50 | LAC, EF, EFL | [54] |
| Gassericin T |  | 57 |  | [55] |
| Halocin C8 |  | 76 |  | [56] |
| Halocin S8 |  | 36 |  | [57] |
| Lactococcin 972 |  | 66 |  | [58] |
| Lactococcin A |  | 54 | LAC | [59] |
| Lactococcin B |  | 47 | LAC | [60] |
| Laterosporulin |  | 50 |  | [61] |
| Leucocin B |  | 31 |  | [11] |
| Leucocin K7 |  | 34 |  | [62] |
| LsbA |  | 44 | LAC | [63] |
| LsbB |  | 30 | LAC | [63] |
| **Bacteriocin** | **Class** | **Size in AA** | **Spectrum of Activity^a,b^** | **Reference^c^** |
| Microcin 24 | IId | 74 | LAC | [64] |
| Microcin B17 (1-41)/Colicin V (57-88) |  | 75 |  | [65] |
| Microcin E492 |  | 84 |  | [66] |
| Microcin H47 (1-51)/Colicin V (57-88) |  | 84 | EC | [65] |
| Microcin H47 (1-60)/Colicin V (57-88) |  | 94 | EC | [65] |
| Microcin L |  | 90 | EC | [67] |
| Microcin M |  | 77 |  | [68] |
| Plantaricin 1,25β |  | 53 |  | [69] |
| Plantaricin A |  | 23 |  | [70] |
| Plantaricin ASM1 |  | 44 |  | [71] |
| Plantaricin N |  | 30 |  | [72] |
| Plantaricin ZJ5 |  | 22 |  | [73] |
| Pneumocin BlpD |  | 36 |  | [74] |
| Pneumocin BlpK |  | 52 | LAC | [74] |
| Propionicin F |  | 43 |  | [75] |
| Propionicin T1 |  | 65 |  | [76] |
| Sakacin Q |  | 49 |  | [77] |
| Salivaricin V |  | 53 |  | WP_002884558.1 |
| Salivaricin W |  | 28 |  | AEJ52683.1 |
| Salivaricin X |  | 43 |  | ALR80677.1 |
| Salivaricin Y |  | 34 |  | WP_045771794.1 |
| Salivaricin Z |  | 33 |  | WP_002887002.1 |
| Subtilosin A |  | 43 |  | [78] |
| Subtilosin X |  | 50 |  | [79] |
| Thuricin 17 |  | 31 |  | [80] |
| Thuricin S |  | 18 |  | [81] |
| Warnericin RK |  | 22 |  | [82] |
| Weissellicin 110 |  | 31 | LAC | [83] |
| Albusin B | III | 290 |  | [84] |
| Alveicin A |  | 408 |  | [85] |
| Bacteriocin 28B |  | 449 | EC | [86] |
| Bacteriocin BCN 5 |  | 890 |  | [87] |
| Carocin D |  | 828 |  | [88] |
| Carocin D - Variant 2 |  | 480 |  | ACA70312.1 |
| Closticin 574 |  | 283 | LAC, EFL | [89] |
| Colicin B |  | 511 | EC | [90] |
| Colicin FY |  | 438 |  | [91] |
| Colicin K |  | 548 | EC | [92] |
| Colicin Y |  | 604 |  | [93] |
| Dysgalacticin |  | 193 |  | [94] |
| **Bacteriocin** | **Class** | **Size in AA** | **Spectrum of Activity^a,b^** | **Reference^c^** |
| Enterolysin A | III | 316 | EF, EFL | [95] |
| Halocin H4 |  | 313 |  | [96] |
| Ipomicin |  | 96 | LAC | [97] |
| Linocin M18 |  | 267 |  | [98] |
| Maritimacin |  | 255 |  | [99] |
| Microcin S |  | 102 |  | [100] |
| Pesticin |  | 358 |  | [101] |
| Propionicin SM1 |  | 180 |  | [102] |
| Putidacin L1 |  | 276 |  | [103] |
| Pyocin S2 |  | 690 |  | [104] |
| Salmocin E1A |  | 483 | BS, BC | [105] |
| Salmocin E1B |  | 527 | EC, BS, BC | [105] |
| Zoocin A |  | 263 |  | [106] |

^a^Bacteriocins that did not show activity against the indicators tested have been left blank since they could be active against an indicator that has not yet been tested.

^b^Strains used in this study as activity indicators: BC, *Bacillus cereus* ATCC 14579; BS, *B. subtilis* 168; EC, *Escherichia coli* DH10B; EFL, *Enterococcus faecalis* Si0159; EF, *E. faecium* ATCC 19434; LAC, *Lactococcus lactis* IL1403; LIS, *Listeria monocytogenes* ATCC 19115; PP, *Pediococcus pentosaceus* HELA; SA, *Staphylococcus aureus aureus* ATCC 6538; SE, *S. epirdermis* ATCC 12228; SM, *Streptococcus mutans* UA159; SP, *S. pyogenes* ATCC 12344.

^c^When a bacteriocin has not been published, the NCBI reference sequence or GenBank number is provided instead.

REFERENCES

1. Kanatani, K., M. Oshimura, and K. Sano, *Isolation and characterization of acidocin A and cloning of the bacteriocin gene from Lactobacillus acidophilus.* Appl Environ Microbiol, 1995. **61**(3): p. 1061-7.

2. Birri, D.J., et al., *Molecular and genetic characterization of a novel bacteriocin locus in Enterococcus avium isolates from infants.* Appl Environ Microbiol, 2010. **76**(2): p. 483-92.

3. Todokoro, D., et al., *Genetic analysis of bacteriocin 43 of vancomycin-resistant Enterococcus faecium.* Appl Environ Microbiol, 2006. **72**(11): p. 6955-64.

4. Huot, E., et al., *Bacteriocin J46, a New Bacteriocin Produced byLactococcus lactisSubsp.cremorisJ46: Isolation and Characterization of the Protein and Its Gene.* Anaerobe, 1996. **2**(3): p. 137-145.

5. Quadri, L.E., et al., *Chemical and genetic characterization of bacteriocins produced by Carnobacterium piscicola LV17B.* J Biol Chem, 1994. **269**(16): p. 12204-11.

6. Farias, M.E., et al., *Purification and N-terminal amino acid sequence of Enterocin CRL 35, a 'pediocin-like' bacteriocin produced by Enterococcus faecium CRL 35.* Lett Appl Microbiol, 1996. **22**(6): p. 417-9.

7. Aymerich, T., et al., *Biochemical and genetic characterization of enterocin A from Enterococcus faecium, a new antilisterial bacteriocin in the pediocin family of bacteriocins.* Appl Environ Microbiol, 1996. **62**(5): p. 1676-82.

8. Svetoch, E.A., et al., *Diverse antimicrobial killing by Enterococcus faecium E 50-52 bacteriocin.* J Agric Food Chem, 2008. **56**(6): p. 1942-8.

9. Line, J.E., et al., *Isolation and purification of enterocin E-760 with broad antimicrobial activity against gram-positive and gram-negative bacteria.* Antimicrob Agents Chemother, 2008. **52**(3): p. 1094-100.

10. Hastings, J.W., et al., *Characterization of leucocin A-UAL 187 and cloning of the bacteriocin gene from Leuconostoc gelidum.* J Bacteriol, 1991. **173**(23): p. 7491-500.

11. Papathanasopoulos, M.A., et al., *Sequence and structural relationships of leucocins A-, B- and C-TA33a from Leuconostoc mesenteroides TA33a.* Microbiology, 1998. **144 ( Pt 5)**: p. 1343-8.

12. Feng, G., et al., *Characterization of mundticin L, a class IIa anti-Listeria bacteriocin from Enterococcus mundtii CUGF08.* Appl Environ Microbiol, 2009. **75**(17): p. 5708-13.

13. Nieto Lozano, J.C., et al., *Purification and amino acid sequence of a bacteriocin produced by Pediococcus acidilactici.* J Gen Microbiol, 1992. **138**(9): p. 1985-90.

14. Tiwari, S.K., et al., *Improved antimicrobial activities of synthetic-hybrid bacteriocins designed from enterocin E50-52 and pediocin PA-1.* Appl Environ Microbiol, 2015. **81**(5): p. 1661-7.

15. Jack, R.W., et al., *Characterization of the chemical and antimicrobial properties of piscicolin 126, a bacteriocin produced by Carnobacterium piscicola JG126.* Appl Environ Microbiol, 1996. **62**(8): p. 2897-903.

16. Van Reenen, C.A., et al., *Characterization and heterologous expression of a class IIa bacteriocin, plantaricin 423 from Lactobacillus plantarum 423, in Saccharomyces cerevisiae.* Int J Food Microbiol, 2003. **81**(1): p. 29-40.

17. Holck, A., et al., *Purification and amino acid sequence of sakacin A, a bacteriocin from Lactobacillus sake Lb706.* J Gen Microbiol, 1992. **138**(12): p. 2715-20.

18. Simon, L., et al., *Sakacin g, a new type of antilisterial bacteriocin.* Appl Environ Microbiol, 2002. **68**(12): p. 6416-20.

19. Tichaczek, P.S., R.F. Vogel, and W.P. Hammes, *Cloning and sequencing of sakP encoding sakacin P, the bacteriocin produced by Lactobacillus sake LTH 673.* Microbiology, 1994. **140 ( Pt 2)**: p. 361-7.

20. Johnsen, L., G. Fimland, and J. Nissen-Meyer, *The C-terminal domain of pediocin-like antimicrobial peptides (class IIa bacteriocins) is involved in specific recognition of the C-terminal part of cognate immunity proteins and in determining the antimicrobial spectrum.* J Biol Chem, 2005. **280**(10): p. 9243-50.

21. Vaughan, A., V.G. Eijsink, and D. Van Sinderen, *Functional characterization of a composite bacteriocin locus from malt isolate Lactobacillus sakei 5.* Appl Environ Microbiol, 2003. **69**(12): p. 7194-203.

22. Heng, N.C., et al., *Ubericin A, a class IIa bacteriocin produced by Streptococcus uberis.* Appl Environ Microbiol, 2007. **73**(23): p. 7763-6.

23. Flynn, S., et al., *Characterization of the genetic locus responsible for the production of ABP-118, a novel bacteriocin produced by the probiotic bacterium Lactobacillus salivarius subsp. salivarius UCC118.* Microbiology, 2002. **148**(Pt 4): p. 973-84.

24. Contreras, B.G., et al., *Isolation, purification, and amino acid sequence of lactobin A, one of the two bacteriocins produced by Lactobacillus amylovorus LMG P-13139.* Appl Environ Microbiol, 1997. **63**(1): p. 13-20.

25. McCormick, J.K., et al., *Genetic characterization and heterologous expression of brochocin-C, an antibotulinal, two-peptide bacteriocin produced by Brochothrix campestris ATCC 43754.* Appl Environ Microbiol, 1998. **64**(12): p. 4757-66.

26. Fremaux, C., C. Ahn, and T.R. Klaenhammer, *Molecular analysis of the lactacin F operon.* Appl Environ Microbiol, 1993. **59**(11): p. 3906-15.

27. Cuozzo, S.A., et al., *Identification and nucleotide sequence of genes involved in the synthesis of lactocin 705, a two-peptide bacteriocin from Lactobacillus casei CRL 705.* FEMS Microbiol Lett, 2000. **185**(2): p. 157-61.

28. Moll, G., et al., *Lactococcin G is a potassium ion-conducting, two-component bacteriocin.* J Bacteriol, 1996. **178**(3): p. 600-5.

29. Zendo, T., et al., *Lactococcin Q, a novel two-peptide bacteriocin produced by Lactococcus lactis QU 4.* Appl Environ Microbiol, 2006. **72**(5): p. 3383-9.

30. Diep, D.B., L.S. Havarstein, and I.F. Nes, *A bacteriocin-like peptide induces bacteriocin synthesis in Lactobacillus plantarum C11.* Mol Microbiol, 1995. **18**(4): p. 631-9.

31. Maldonado, A., J.L. Ruiz-Barba, and R. Jimenez-Diaz, *Purification and genetic characterization of plantaricin NC8, a novel coculture-inducible two-peptide bacteriocin from Lactobacillus plantarum NC8.* Appl Environ Microbiol, 2003. **69**(1): p. 383-9.

32. Jimenez-Diaz, R., et al., *Purification and partial amino acid sequence of plantaricin S, a bacteriocin produced by Lactobacillus plantarum LPCO10, the activity of which depends on the complementary action of two peptides.* Appl Environ Microbiol, 1995. **61**(12): p. 4459-63.

33. Holo, H., et al., *Plantaricin W from Lactobacillus plantarum belongs to a new family of two-peptide lantibiotics.* Microbiology, 2001. **147**(Pt 3): p. 643-51.

34. Marciset, O., et al., *Thermophilin 13, a nontypical antilisterial poration complex bacteriocin, that functions without a receptor.* J Biol Chem, 1997. **272**(22): p. 14277-84.

35. Rea, M.C., et al., *Thuricin CD, a posttranslationally modified bacteriocin with a narrow spectrum of activity against Clostridium difficile.* Proc Natl Acad Sci U S A, 2010. **107**(20): p. 9352-7.

36. Netz, D.J., C. Bastos Mdo, and H.G. Sahl, *Mode of action of the antimicrobial peptide aureocin A53 from Staphylococcus aureus.* Appl Environ Microbiol, 2002. **68**(11): p. 5274-80.

37. Netz, D.J., et al., *Molecular characterisation of aureocin A70, a multi-peptide bacteriocin isolated from Staphylococcus aureus.* J Mol Biol, 2001. **311**(5): p. 939-49.

38. Ovchinnikov, K.V., et al., *Novel Group of Leaderless Multipeptide Bacteriocins from Gram-Positive Bacteria.* Appl Environ Microbiol, 2016. **82**(17): p. 5216-24.

39. Liu, X., et al., *Identification of an N-terminal formylated, two-peptide bacteriocin from Enterococcus faecalis 710C.* J Agric Food Chem, 2011. **59**(10): p. 5602-8.

40. Cintas, L.M., et al., *Enterocins L50A and L50B, two novel bacteriocins from Enterococcus faecium L50, are related to staphylococcal hemolysins.* J Bacteriol, 1998. **180**(8): p. 1988-94.

41. Fujita, K., et al., *Structural analysis and characterization of lacticin Q, a novel bacteriocin belonging to a new family of unmodified bacteriocins of gram-positive bacteria.* Appl Environ Microbiol, 2007. **73**(9): p. 2871-7.

42. Iwatani, S., et al., *Characterization and structure analysis of a novel bacteriocin, lacticin Z, produced by Lactococcus lactis QU 14.* Biosci Biotechnol Biochem, 2007. **71**(8): p. 1984-92.

43. Hyink, O., M. Balakrishnan, and J.R. Tagg, *Streptococcus rattus strain BHT produces both a class I two-component lantibiotic and a class II bacteriocin.* FEMS Microbiol Lett, 2005. **252**(2): p. 235-41.

44. Masuda, Y., et al., *Characterization and identification of weissellicin Y and weissellicin M, novel bacteriocins produced by Weissella hellenica QU 13.* J Appl Microbiol, 2012. **112**(1): p. 99-108.

45. Kanatani, K., et al., *Cloning and nucleotide sequence of the gene for acidocin 8912, a bacteriocin from Lactobacillus acidophilus TK8912.* Lett Appl Microbiol, 1995. **21**(6): p. 384-6.

46. Inoue, T., H. Tomita, and Y. Ike, *Bac 32, a novel bacteriocin widely disseminated among clinical isolates of Enterococcus faecium.* Antimicrob Agents Chemother, 2006. **50**(4): p. 1202-12.

47. Garnier, T. and S.T. Cole, *Complete nucleotide sequence and genetic organization of the bacteriocinogenic plasmid, pIP404, from Clostridium perfringens.* Plasmid, 1988. **19**(2): p. 134-50.

48. O'Shea, E.F., et al., *Bactofencin A, a new type of cationic bacteriocin with unusual immunity.* MBio, 2013. **4**(6): p. e00498-13.

49. Oscáriz, J.C., et al., *Purification and sequencing of cerein 7B, a novel bacteriocin produced by Bacillus cereus Bc7.* FEMS Microbiology Letters, 2006. **254**(1): p. 108-115.

50. Gratia, A., *Sur un remarquable exemple d'antagonisme entre deux souches de colibacille.* C R Soc Biol, 1925. **93**: p. 1040–1042.

51. Galvez, A., et al., *Isolation and characterization of enterocin EJ97, a bacteriocin produced by Enterococcus faecalis EJ97.* Arch Microbiol, 1998. **171**(1): p. 59-65.

52. Ovchinnikov, K.V., et al., *The Leaderless Bacteriocin Enterocin K1 Is Highly Potent against Enterococcus faecium: A Study on Structure, Target Spectrum and Receptor.* Front Microbiol, 2017. **8**: p. 774.

53. Sandiford, S. and M. Upton, *Identification, characterization, and recombinant expression of epidermicin NI01, a novel unmodified bacteriocin produced by Staphylococcus epidermidis that displays potent activity against Staphylococci.* Antimicrob Agents Chemother, 2012. **56**(3): p. 1539-47.

54. Tosukhowong, A., et al., *Garvieacin Q, a novel class II bacteriocin from Lactococcus garvieae BCC 43578.* Appl Environ Microbiol, 2012. **78**(5): p. 1619-23.

55. Kawai, Y., et al., *Primary amino acid and DNA sequences of gassericin T, a lactacin F-family bacteriocin produced by Lactobacillus gasseri SBT2055.* Biosci Biotechnol Biochem, 2000. **64**(10): p. 2201-8.

56. Li, Y., et al., *Purification and biological characterization of halocin C8, a novel peptide antibiotic from Halobacterium strain AS7092.* Extremophiles, 2003. **7**(5): p. 401-7.

57. Price, L.B. and R.F. Shand, *Halocin S8: a 36-amino-acid microhalocin from the haloarchaeal strain S8a.* J Bacteriol, 2000. **182**(17): p. 4951-8.

58. Martinez, B., et al., *Synthesis of lactococcin 972, a bacteriocin produced by Lactococcus lactis IPLA 972, depends on the expression of a plasmid-encoded bicistronic operon.* Microbiology, 1999. **145 ( Pt 11)**: p. 3155-61.

59. Holo, H., O. Nilssen, and I.F. Nes, *Lactococcin A, a new bacteriocin from Lactococcus lactis subsp. cremoris: isolation and characterization of the protein and its gene.* J Bacteriol, 1991. **173**(12): p. 3879-87.

60. van Belkum, M.J., J. Kok, and G. Venema, *Cloning, sequencing, and expression in Escherichia coli of lcnB, a third bacteriocin determinant from the lactococcal bacteriocin plasmid p9B4-6.* Appl Environ Microbiol, 1992. **58**(2): p. 572-7.

61. Singh, P.K., et al., *Identification, purification and characterization of laterosporulin, a novel bacteriocin produced by Brevibacillus sp. strain GI-9.* PLoS One, 2012. **7**(3): p. e31498.

62. Shi, F., et al., *Mode of action of leucocin K7 produced by Leuconostoc mesenteroides K7 against Listeria monocytogenes and its potential in milk preservation.* Biotechnol Lett, 2016. **38**(9): p. 1551-7.

63. Gajic, O., et al., *Novel mechanism of bacteriocin secretion and immunity carried out by lactococcal multidrug resistance proteins.* J Biol Chem, 2003. **278**(36): p. 34291-8.

64. O'Brien, G.J. and H.K. Mahanty, *Colicin 24, a new plasmid-borne colicin from a uropathogenic strain of Escherichia coli.* Plasmid, 1994. **31**(3): p. 288-96.

65. Azpiroz, M.F. and M. Lavina, *Modular structure of microcin H47 and colicin V.* Antimicrob Agents Chemother, 2007. **51**(7): p. 2412-9.

66. de Lorenzo, V., *Isolation and characterization of microcin E492 from Klebsiella pneumoniae.* Arch Microbiol, 1984. **139**(1): p. 72-5.

67. Gaillard-Gendron, S., et al., *Isolation, purification and partial amino acid sequence of a highly hydrophobic new microcin named microcin L produced by Escherichia coli.* FEMS Microbiol Lett, 2000. **193**(1): p. 95-8.

68. Vassiliadis, G., et al., *Microcin M, an antibacterial peptide from the probiotic bacterium Escherichia coli Nissle 1917*, in *1st International Symposium Food, Veterinary and Medical Applications of Antimicrobial Peptides*. 2006: ENITIAA, Nantes, France.

69. Ehrmann, M.A., et al., *A gene cluster encoding plantaricin 1.25beta and other bacteriocin-like peptides in Lactobacillus plantarum TMW1.25.* Biochim Biophys Acta, 2000. **1490**(3): p. 355-61.

70. Nissen-Meyer, J., et al., *Purification and characterization of plantaricin A, a Lactobacillus plantarum bacteriocin whose activity depends on the action of two peptides.* J Gen Microbiol, 1993. **139**(9): p. 1973-8.

71. Hata, T., R. Tanaka, and S. Ohmomo, *Isolation and characterization of plantaricin ASM1: a new bacteriocin produced by Lactobacillus plantarum A-1.* Int J Food Microbiol, 2010. **137**(1): p. 94-9.

72. Diep, D.B., L.S. Havarstein, and I.F. Nes, *Characterization of the locus responsible for the bacteriocin production in Lactobacillus plantarum C11.* J Bacteriol, 1996. **178**(15): p. 4472-83.

73. Song, D.F., M.Y. Zhu, and Q. Gu, *Purification and characterization of Plantaricin ZJ5, a new bacteriocin produced by Lactobacillus plantarum ZJ5.* PLoS One, 2014. **9**(8): p. e105549.

74. Bogaardt, C., A.J. van Tonder, and A.B. Brueggemann, *Genomic analyses of pneumococci reveal a wide diversity of bacteriocins - including pneumocyclicin, a novel circular bacteriocin.* BMC Genomics, 2015. **16**: p. 554.

75. Brede, D.A., et al., *Molecular and genetic characterization of propionicin F, a bacteriocin from Propionibacterium freudenreichii.* Appl Environ Microbiol, 2004. **70**(12): p. 7303-10.

76. Faye, T., et al., *Biochemical and genetic characterization of propionicin T1, a new bacteriocin from Propionibacterium thoenii.* Appl Environ Microbiol, 2000. **66**(10): p. 4230-6.

77. Mathiesen, G., et al., *Characterization of a new bacteriocin operon in sakacin P-producing Lactobacillus sakei, showing strong translational coupling between the bacteriocin and immunity genes.* Appl Environ Microbiol, 2005. **71**(7): p. 3565-74.

78. Babasaki, K., et al., *Subtilosin A, a new antibiotic peptide produced by Bacillus subtilis 168: isolation, structural analysis, and biogenesis.* J Biochem, 1985. **98**(3): p. 585-603.

79. Zheng, G., R. Hehn, and P. Zuber, *Mutational analysis of the sbo-alb locus of Bacillus subtilis: identification of genes required for subtilosin production and immunity.* J Bacteriol, 2000. **182**(11): p. 3266-73.

80. Gray, E.J., et al., *A novel bacteriocin, thuricin 17, produced by plant growth promoting rhizobacteria strain Bacillus thuringiensis NEB17: isolation and classification.* J Appl Microbiol, 2006. **100**(3): p. 545-54.

81. Chehimi, S., et al., *Purification and partial amino acid sequence of thuricin S, a new anti-Listeria bacteriocin from Bacillus thuringiensis.* Can J Microbiol, 2007. **53**(2): p. 284-90.

82. Verdon, J., et al., *Characterization of anti-Legionella activity of warnericin RK and delta-lysin I from Staphylococcus warneri.* Peptides, 2008. **29**(6): p. 978-84.

83. Srionnual, S., et al., *Weissellicin 110, a newly discovered bacteriocin from Weissella cibaria 110, isolated from plaa-som, a fermented fish product from Thailand.* Appl Environ Microbiol, 2007. **73**(7): p. 2247-50.

84. Chen, J., D.M. Stevenson, and P.J. Weimer, *Albusin B, a Bacteriocin from the Ruminal Bacterium <em>Ruminococcus albus</em> 7 That Inhibits Growth of <em>Ruminococcus flavefaciens</em>.* 2004. **70**(5): p. 3167-3170.

85. Wertz, J.E. and M.A. Riley, *Chimeric nature of two plasmids of Hafnia alvei encoding the bacteriocins alveicins A and B.* J Bacteriol, 2004. **186**(6): p. 1598-605.

86. Guasch, J.F., et al., *Bacteriocin 28b, a chromosomally encoded bacteriocin produced by most Serratia marcescens biotypes.* Res Microbiol, 1995. **146**(6): p. 477-83.

87. Garnier, T. and S.T. Cole, *Characterization of a bacteriocinogenic plasmid from Clostridium perfringens and molecular genetic analysis of the bacteriocin-encoding gene.* J Bacteriol, 1986. **168**(3): p. 1189-96.

88. Roh, E., et al., *Characterization of a new bacteriocin, Carocin D, from Pectobacterium carotovorum subsp. carotovorum Pcc21.* Appl Environ Microbiol, 2010. **76**(22): p. 7541-9.

89. Kemperman, R., et al., *Identification and characterization of two novel clostridial bacteriocins, circularin A and closticin 574.* Appl Environ Microbiol, 2003. **69**(3): p. 1589-97.

90. Schramm, E., et al., *Nucleotide sequence of the colicin B activity gene cba: consensus pentapeptide among TonB-dependent colicins and receptors.* J Bacteriol, 1987. **169**(7): p. 3350-7.

91. Bosak, J., et al., *Novel colicin Fy of Yersinia frederiksenii inhibits pathogenic Yersinia strains via YiuR-mediated reception, TonB import, and cell membrane pore formation.* J Bacteriol, 2012. **194**(8): p. 1950-9.

92. Goebel, W.F., *The nature of the colicin K of Escherichia coli K235.* Proc Natl Acad Sci U S A, 1973. **70**(3): p. 854-8.

93. Riley, M.A., et al., *The newly characterized colicin Y provides evidence of positive selection in pore-former colicin diversification.* Microbiology, 2000. **146 ( Pt 7)**: p. 1671-7.

94. Heng, N.C., et al., *Dysgalacticin: a novel, plasmid-encoded antimicrobial protein (bacteriocin) produced by Streptococcus dysgalactiae subsp. equisimilis.* Microbiology, 2006. **152**(Pt 7): p. 1991-2001.

95. Nilsen, T., I.F. Nes, and H. Holo, *Enterolysin A, a cell wall-degrading bacteriocin from Enterococcus faecalis LMG 2333.* Appl Environ Microbiol, 2003. **69**(5): p. 2975-84.

96. Cheung, J., et al., *Isolation, sequence, and expression of the gene encoding halocin H4, a bacteriocin from the halophilic archaeon Haloferax mediterranei R4.* J Bacteriol, 1997. **179**(2): p. 548-51.

97. Wang, J., K.L. Schully, and G.S. Pettis, *Growth-regulated expression of a bacteriocin, produced by the sweet potato pathogen Streptomyces ipomoeae, that exhibits interstrain inhibition.* Appl Environ Microbiol, 2009. **75**(5): p. 1236-42.

98. Valdes-Stauber, N. and S. Scherer, *Nucleotide sequence and taxonomical distribution of the bacteriocin gene lin cloned from Brevibacterium linens M18.* Appl Environ Microbiol, 1996. **62**(4): p. 1283-6.

99. Hicks, P.M., et al., *Homomultimeric protease in the hyperthermophilic bacterium Thermotoga maritima has structural and amino acid sequence homology to bacteriocins in mesophilic bacteria.* FEBS Lett, 1998. **440**(3): p. 393-8.

100. Zschuttig, A., et al., *Identification and characterization of microcin S, a new antibacterial peptide produced by probiotic Escherichia coli G3/10.* PLoS One, 2012. **7**(3): p. e33351.

101. Patzer, S.I., et al., *Structural and mechanistic studies of pesticin, a bacterial homolog of phage lysozymes.* J Biol Chem, 2012. **287**(28): p. 23381-96.

102. Miescher, S., et al., *Propionicin SM1, a bacteriocin from Propionibacterium jensenii DF1: isolation and characterization of the protein and its gene.* Syst Appl Microbiol, 2000. **23**(2): p. 174-84.

103. Parret, A.H., et al., *Plant lectin-like bacteriocin from a rhizosphere-colonizing Pseudomonas isolate.* J Bacteriol, 2003. **185**(3): p. 897-908.

104. Sano, Y., et al., *Molecular structures and functions of pyocins S1 and S2 in Pseudomonas aeruginosa.* J Bacteriol, 1993. **175**(10): p. 2907-16.

105. Schneider, T., et al., *Plant-made Salmonella bacteriocins salmocins for control of Salmonella pathovars.* Sci Rep, 2018. **8**(1): p. 4078.

106. Simmonds, R.S., W.J. Simpson, and J.R. Tagg, *Cloning and sequence analysis of zooA, a Streptococcus zooepidemicus gene encoding a bacteriocin-like inhibitory substance having a domain structure similar to that of lysostaphin.* Gene, 1997. **189**(2): p. 255-61.
